# Thickness Effect on CO_2_/N_2_ Separation in Double Layer Pebax-1657^®^/PDMS Membranes

**DOI:** 10.3390/membranes8040121

**Published:** 2018-12-02

**Authors:** Roman Selyanchyn, Miho Ariyoshi, Shigenori Fujikawa

**Affiliations:** 1 WPI International Institute for Carbon-Neutral Energy Research (WPI-I2CNER), Kyushu University, 744 Motooka, Nishi-ku, Fukuoka 819-0395, Japan; ariyoshi.miho.023@m.kyushu-u.ac.jp; 2 NanoMembrane Technologies Inc., 4-1, Kyudai-Shimachi, Nishi-ku, Fukuoka 819-0388, Japan; 3 Center for Molecular Systems (CMS), Kyushu University, 744 Motooka, Nishi-ku, Fukuoka 819-0395, Japan; 4 Laboratory for Chemistry and Life Science, Tokyo Institute of Technology, 4259 Nagatsutacho, Midori-ku, Yokohama 226-8503, Japan

**Keywords:** gas separation, membrane, thickness influence, thin-film nanocomposite membranes, flue gas separation, carbon dioxide capture, carbon-neutral energy

## Abstract

The effect of thickness in multilayer thin-film composite membranes on gas permeation has received little attention to date, and the gas permeances of the organic polymer membranes are believed to increase by membrane thinning. Moreover, the performance of defect-free layers with known gas permeability can be effectively described using the classical resistance in series models to predict both permeance and selectivity of the composite membrane. In this work, we have investigated the Pebax^®^-MH1657/PDMS double layer membrane as a selective/gutter layer combination that has the potential to achieve sufficient CO_2_/N_2_ selectivity and permeance for efficient CO_2_ and N_2_ separation. CO_2_ and N_2_ transport through membranes with different thicknesses of two layers has been investigated both experimentally and with the utilization of resistance in series models. Model prediction for permeance/selectivity corresponded perfectly with experimental data for the thicker membranes. Surprisingly, a significant decrease from model predictions was observed when the thickness of the polydimethylsiloxane (PDMS) (gutter layer) became relatively small (below 2 µm thickness). Material properties changed at low thicknesses—surface treatments and influence of porous support are discussed as possible reasons for observed deviations.

## 1. Introduction

Membranes are considered a potentially effective tool for separation of carbon dioxide (CO_2_) from gas mixtures. In particular, separation from ubiquitous flue-gas originating from heat-power plants is needed to achieve cost-efficient CO_2_ capture and prevent global warming [1]. Membrane systems are viewed as competitive to conventional CO_2_ capture systems due to versatility, relative simplicity, potentially lower energy consumption, and lower capital cost as well as smaller environmental footprint [2]. On the other hand, to succeed in large-scale industrial application, gas separation membranes should satisfy not only high “separation ability” i.e., selectivity towards CO_2_ (e.g., >40 in post-combustion CO_2_/N_2_ separation), but also significantly higher “separation speed” i.e., permeance numbers (>1000 GPU) [3]. A significant number of organic polymer materials have demonstrated suitable values of CO_2_/N_2_ selectivity, well exceeding the number required for industrial use. However, at the same time, gas permeability and selectivity in organic polymers follow well-known trade-off behavior resulting in the so-called Robeson upper bound of performance [4]. According to the empirical data published in literature, most organic polymers with high selectivity have considerably low permeability at the same time. As a result, it is quite difficult to fabricate the membranes with satisfactory outcomes using just one separating layer of material in the membrane. The required thicknesses of such separation layers able to achieve the permeances as high as 4000 GPU for CO_2_ are very thin (<30 nm), and therefore it is unlikely to fabricate such thin films without defects on a large scale [5]. Moreover, a number of negative effects were observed in the layers of such ultimate thickness: accelerated aging, pronounced defect formation, and permeability decrease with thickness [5,6,7]. Thin film composite (TFC) membranes with more complex structures are aiming to overcome these drawbacks. In such membranes, the thin selective layer is most often assembled on a highly gas-permeable polymer layer, the so-called gutter layer, which is previously deposited on the porous support and combined with a mechanical support layer. Utilization of gutter layers provide some advantages to improve the membrane performance. In particular, it is preventing the viscous flow of gas through the minor defects that can be present in the selective layers [8]. Polydimethylsiloxane (PDMS, rubbery polymer) and poly(1-(trimethylsilyl)-1-propyne) (PTMSP, glassy polymer) are two materials most commonly used as gutter layers in TFC membranes, due to their high gas permeability [9]. In recent works, PTMSP of a few micrometer thickness was used as the gutter layer on poly(vinylidene difluoride) (PVDF) to support graphene oxide-ionic liquid [10] or metal-organic framework [11] based selective layers. In contrast, PDMS could be easily used with much smaller thicknesses (down to 100 nm) and has been utilized as a gutter layer on polyacrylonitrile (PAN) porous supports, most often to hold even thinner polymeric or mixed matrix layers [12,13]. However, despite its superior initial bulk permeability, PTMSP demonstrates accelerated aging [14], and therefore PDMS retains almost exclusively used as a gutter layer in the majority of reported TFC membranes for CO_2_ separation [9].

The family of thermoplastic polyether-block-amides commercially available as Pebax^®^ is a class of polymers well known in the membrane research field. These polymers contain ethylene oxide moieties, which are known for their selectivity of CO_2_ over other gases such as nitrogen, methane, and even smaller gases like H_2_ and He [15]. Structurally, these polymers contain polyethylene oxide (PEO) and polyamides nylon-6 or nylon-12 (PA6 and PA12, respectively). Owing to the presence of PA in the structure, Pebax polymers possess excellent mechanical properties while phase-separated PEO domains act as the pathways for the selective gas transport. Within this family, the polymer grade identified as Pebax^®^ MH1657 containing 60 wt% of PEO and 40 wt% polyamide-6 PA6 in its structure is one of the most investigated for selective layer formation in TFC separation membranes for CO_2_ capture [9]. It possesses high selectivity towards CO_2_ in both CO_2_/N_2_ (ca. 50–70), and CO_2_/H_2_ (ca. 10) targeted separations as well as reasonable CO_2_ permeability (~100 barrer at room temperature).

**Figure 1:**
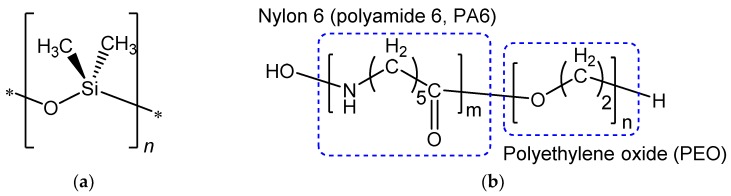
Chemical structures of polymers used for double layer membrane fabrication: (**a**) polydimethylsiloxane (PDMS) and (**b**) general structure of Pebax^®^ polymer family containing different amounts of polyamide 6 and polyethylene oxide blocks in the structure.

In this current work, we investigate the double layer membrane composed of Pebax-1657 selective layer and PDMS gutter layer (chemical structures are given in Figure 1) transferred onto porous support to form a composite membrane. Prediction of the double layer membrane permeance and selectivity are calculated using the resistance in series model [16] based on the bulk permeabilities of both polymers. We have fabricated membranes with various thickness combinations for two layers and compared experimental results with the model prediction.

## 2. Materials and Methods

### 2.1. Materials

Simple soda-lime glass substrates (3.5 × 3.5 cm) were used for double layer membrane fabrication by spin-coating. Polyhydroxystyrene (PHS, Mw = 11,000, Sigma-Aldrich, St. Louis, MO, USA) was used for the fabrication of a sacrificial layer. A two-component PDMS kit (Sylgard184) was purchased from Dow Corning. Copolymer Pebax^®^ MH1657 (Pebax-1657) containing 60 wt% of poly(ethylene oxide) (PEO) and 40 wt% polyamide-6 (PA6) in its structure was provided by Arkema (Kyoto, Japan) in the form of pellets and used as received for membrane preparation. Hexane (a mixture of isomers, ≥96%) and ethanol were purchased from Wako Pure Chemicals (Osaka, Japan) and used as received. Deionized water (18.3 MΩ cm^−1^) was obtained by reverse osmosis followed by ion exchange and filtration (Millipore, Direct-QTM, Burlington, MA, USA) and used for substrate washing and solution preparation. The porous support used in this study showed meager resistance to the CO_2_ and N_2_ flow (permeances of support ~55,000 GPU for CO_2_ and ~65,000 GPU for N_2,_ respectively). Therefore, the effect of the porous support can be neglected from resistance model calculations.

### 2.2. Membrane Fabrication

Thick membranes of both Pebax-1657 and PDMS were fabricated via solution casting onto the Teflon molds. Different PDMS membranes were also fabricated to check the permeability of CO_2_ and N_2_ depending on the curing temperature and curing time. Several thick membranes of Pebax-1657 were fabricated to check the reproducibility of bulk permeability and selectivity values.

**Figure 2:**
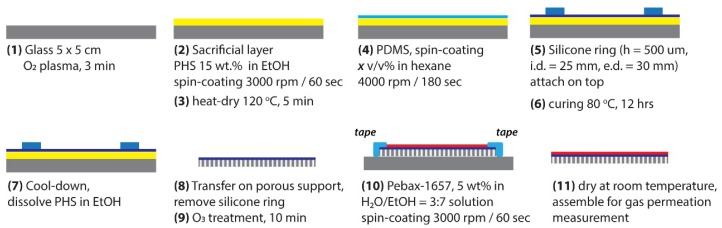
Schematic procedure of the Pebax-1657/PDMS/porous support thin film composite (TFC) membrane fabrication.

Thin film composite (TFC) membranes were fabricated according to previous reports [8,17,18] using the procedure schematically depicted in Figure 2. At first glass substrates were washed in water and ethanol, dried by air blowing, and treated with oxygen plasma for 3 min (FA-1, SAMCO, Kyoto, Japan. RF power: 55 W; flow rate of oxygen: 10 sccm; chamber pressure: 20 Pa) to make the substrate surface hydrophilic. A sacrificial layer (SL) was deposited by spin coating of the 15 wt% PHS solution in EtOH on the plasma treated glass (step 2) followed by drying on the hot-plate for 5 min (step 3). Different thicknesses of PDMS layers were deposited on the glass with an SL layer (step 4) by spin-coating the various concentration solutions of PDMS in hexane (from 1.5 *v*/*v*% to 30 *v*/*v*%). After PDMS spin coating, a silicone ring with a thickness of 500 µm was placed on the PDMS (step 5) and the membrane was cured at 80 °C for 12 h for PDMS crosslinking (step 6). After the completion of the crosslinking, the SL layer was dissolved in EtOH (step 7) to release the freestanding PDMS membrane, which was subsequently removed from EtOH and transferred onto porous support (step 8). For the Pebax-1657 deposition, double layer PDMS/porous support membrane was again physically attached to the glass substrate using adhesive tape. To achieve good adherence of the Pebax-1657 layer to PDMS, a 10 min ozone treatment of the PDMS surface was essential (step 9). Pebax-1657 was spin-coated by using 5 *v*/*v*% solution of the polymer in EtOH/H_2_O (70/30 wt%) mixed solvent (step 10). Finally, the tri-layer TFC membrane was dried (step 11) and was then ready for gas separation tests.

Morphology of the membrane cross-sections was analyzed using field emission scanning electron microscopy (FESEM Hitachi S-5200, 5 kV accelerating voltage). For cross-section observation, the samples were fractured in liquid nitrogen and dried under vacuum. To prevent charge-up by electron beam, samples were coated with a thin platinum layer before observation, deposited using an ion sputter (Hitachi E-1030).

### 2.3. Gas Permeation Measurement

For the gas permeance measurements membranes were masked with aluminum tape to provide the circle of desired diameter and area (*d* = 2 cm, *S* = 3.14 cm^2^, casted membranes; *d* = 1 cm, *S* = 0.79 cm^2^, TFC membranes). The dry gas (N_2_, CO_2_) permeation through the membranes was measured at 25 °C using the GTR-11A/31A gas permeation analysis system (GTR Tec Corp., Kyoto, Japan, operating according to ISO15105-1 B standard) able to measure wide ranges of permeance from very low to moderate values [19]. This system employs a differential-pressure method for testing film permeability with initial vacuum on the permeate side and compressed gas at the feed side, respectively. Total pressure difference was set to 0.2 MPa for all measurements. The sample collection time after vacuuming the permeate side of the membrane was set to 10 min for thick membranes and below 30 s for TFC membranes. The machine is able to measure both pure gas permeability and mixed gas blends as well. In both cases gas chromatograph (GC) is used to measure the amount of permeated gas or gases. The gas collected in the permeation cell is automatically transferred to a GC (Yanaco G3700T, Fukuoka, Japan) which is equipped with a thermal conductivity detector (TCD), and He was used as a carrier gas for sample transport into the machine. Permeance (*P*) of gases in GPU units (1 GPU = 7.5 × 10^−12^ m^3^(STP)/m^2^·s·Pa) was estimated for membranes of different composition and permeability (*p*) in barrer units (1 barrer = 10^−10^ cm^3^(STP)·cm/cm^2^·s·cmHg = GPU·µm) was calculated using the membrane thicknesses and permeances (*p* = *P·l*). The ideal selectivity between two different gases in a hybrid membrane was calculated using the ratio of the permeabilities of a single gas, *α*_A/B_= *p*_A_/*p*_B_, where *p*_A_ and *p*_B_ are the permeabilities of gases A and B, respectively.

### 2.4. Resistance Model for Gas Permeation Description on Three-Layer System

**Figure 3:**
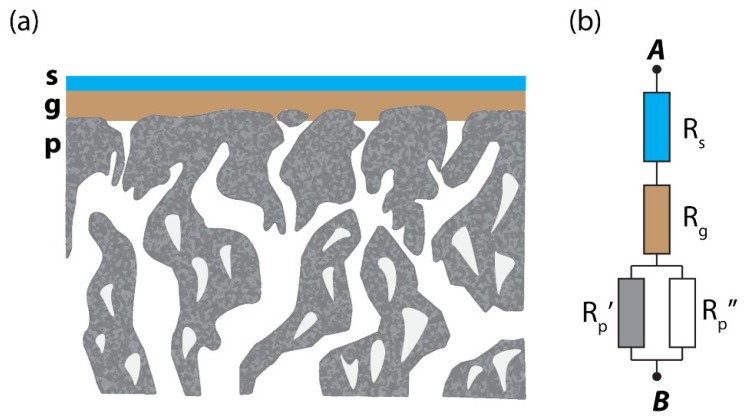
(**a**) General structure of TFC membranes investigated in this work consisting of three layers: **s**—selective (Pebax-1657), **g**—gutter (PDMS), and **p**—porous support layer. (**b**) Electrical circuit analog of the tri-layer structure.

In this research, the composite membranes consist of three layers: the selective layer (Pebax-1657), the gutter layer (PDMS), and porous substrate as schematically shown in Figure 3. According to the resistance in series model [16], gas transport of a layered membrane can be described as an analogy to the electric current flow in the serial connection of conductors. Each layer provides certain resistance to the gas flow which is proportional to the thickness and inversely proportional to the material permeability and surface area (Equation (1)). In the case of the multi-layer structure, the total resistance will be equal to the sum of resistances originating from each layer (Equation (2)). Therefore, the knowledge of mass transport through each layer separately enables one to predict the gas flow through the composite structure. In many cases, and in this work in particular, the resistance of the porous support layer (*R_p_*) can be neglected so that we can consider only the contribution of the selective layer, *R_s_* (Pebax-1657), and gutter layer, *R_g_* (PDMS).

(1) $$R=\frac{l}{p\cdot S}$$

(2) $$R_{t}=R_{s}+R_{g}+R_{p}\approx R_{s}+R_{g}$$

(3) $$\frac{l_{t}}{p_{t}\cdot S}=\frac{l_{s}}{p_{s}\cdot S}+\frac{l_{g}}{p_{g}\cdot S}$$

(4) $$p_{t}=\frac{\left(l_{s}+l_{g}\right)\cdot p_{s}\cdot p_{g}}{p_{g}\cdot l_{s}+p_{s}\cdot l_{g}}$$

Using Equations (3) and (4), it is possible, in principle, to design a membrane with the desired permeability (*p_t_*) and selectivity. In this work, we have considered the CO_2_ and N_2_ separation with the double layer Pebax-1657/PDMS membrane because neither of the materials can satisfy the optimal CO_2_/N_2_ separation due to trade-off phenomena [4]. Namely, being sufficiently selective to CO_2_ (*α* ≈ 64), Pebax-1657 has low CO_2_ permeability (~100 barrer). In contrast, for PDMS the situation is opposite—CO_2_ permeability of the polymer is sufficiently high (~3000 barrer), but rather low selectivity (*α* ≈ 11) is not sufficient for effective CO_2_/N_2_ separation. The permeability of carbon dioxide and nitrogen of the double layer structure will be described by Equations (5) and (6), respectively. The ideal selectivity also can be derived by taking the ratio of permeabilities and can be calculated using Equation (7).

(5) $$p_{t}^{{\mathrm{CO}}_{2}}=\frac{\left(l_{s}+l_{g}\right)\cdot p_{s}^{{\mathrm{CO}}_{2}}\cdot p_{g}^{{\mathrm{CO}}_{2}}}{p_{g}^{{\mathrm{CO}}_{2}}\cdot l_{s}+p_{s}^{{\mathrm{CO}}_{2}}\cdot l_{g}}$$

(6) $$p_{t}^{N_{2}}=\frac{\left(l_{s}+l_{g}\right)\cdot p_{s}^{N_{2}}\cdot p_{g}^{N_{2}}}{p_{g}^{N_{2}}\cdot l_{s}+p_{s}^{N_{2}}\cdot l_{g}}$$

(7) $$\alpha_{{\mathrm{CO}}_{2}{/N}_{2}}=\alpha_{s}\cdot \alpha_{g}\frac{p_{g}^{N_{2}}\cdot l_{s}+p_{s}^{N_{2}}\cdot l_{g}}{p_{g}^{{\mathrm{CO}}_{2}}\cdot l_{s}+p_{s}^{{\mathrm{CO}}_{2}}\cdot l_{g}}$$

where $p_{s}^{{\mathrm{CO}}_{2}},\;p_{s}^{N_{2}},\;\alpha_{s}$ are CO_2_ permeability, N_2_ permeability, and selectivity of selective (Pebax-1657) layer, respectively; $p_{g}^{{\mathrm{CO}}_{2}},\;p_{g}^{N_{2}},\;\alpha_{g}$ are CO_2_ permeability, N_2_ permeability, and selectivity of gutter (PDMS) layer respectively; $p_{t}^{{\mathrm{CO}}_{2}},\;p_{t}^{N_{2}},\;\alpha_{{\mathrm{CO}}_{2}/N_{2}}$ are predicted CO_2_, N_2_ permeabilities, and selectivity of composite membrane.

Further generalization of the Equations (5)–(7) can be done with the consideration of the ratio between the thicknesses of the gutter and selective layer. If we consider *n* = *l_g_*/*l_s_* as a ratio of thicknesses, equations can be rewritten as follows

(8) $$p_{t}^{{\mathrm{CO}}_{2}}=\frac{\left(1+n\right)\cdot p_{s}^{{\mathrm{CO}}_{2}}\cdot p_{g}^{{\mathrm{CO}}_{2}}}{p_{g}^{{\mathrm{CO}}_{2}}+p_{s}^{{\mathrm{CO}}_{2}}\cdot n}$$

(9) $$\alpha_{{\mathrm{CO}}_{2}{/N}_{2}}=\alpha_{s}^{}\cdot \alpha_{g}^{}\frac{p_{g}^{N_{2}}+p_{s}^{N_{2}}\cdot n}{p_{g}^{{\mathrm{CO}}_{2}}+p_{s}^{{\mathrm{CO}}_{2}}\cdot n}$$

Using these two equations, we are generally free to choose the parameters of the double layer membrane to achieve the desired resulting CO_2_ permeability and CO_2_/N_2_ selectivity by varying the thickness ratio between two layers with known gas separation parameters. This work aims to check how close a model prediction would be to the experimental results depending on the thicknesses of the two layers.

## 3. Results

### 3.1. Confirmation of the Bulk Permeability Values of PDMS and Pebax-1657

**Figure 4:**
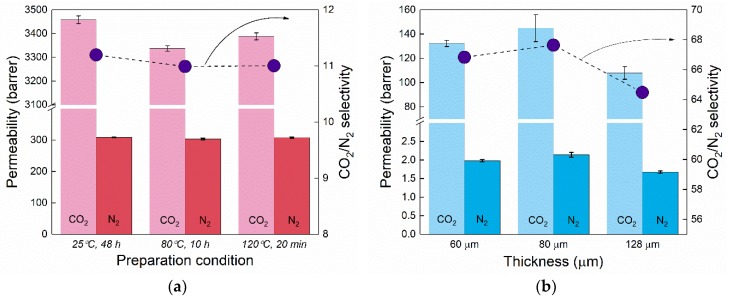
(**a**) Permeability of CO_2_, N_2,_ and CO_2_/N_2_ selectivity in bulk casted PDMS membranes crosslinked in different temperature conditions. (**b**) Permeability of CO_2_, N_2,_ and CO_2_/N_2_ selectivity in bulk Pebax-1657 films with different thicknesses fabricated via solution casting.

Thick membranes of both polymers were fabricated via solution casting and their permeability towards pure gases was checked using GTR-11A/31A equipment. Due to utilization of rather mild curing temperatures for thin PDMS membranes (80 °C), we first tested how this could affect the bulk PDMS permeability. A recent study has reported that the amount of crosslinker and the PDMS curing temperature can have significant influence on CO_2_ permeability [20]. However, we did not detect any significant permeability change with three different PDMS curing temperatures. As seen in Figure 4a, no influence of the PDMS cross-linking temperature on the CO_2_ and N_2_ permeability was detected and materials with different fabrication conditions gave similar gas transport rates. These values of both CO_2_ and N_2_ permeabilities are in good agreement with the reported values in the literature for PDMS [21,22,23,24], considering the measurement temperature. In the case of Pebax-1657, the permeability with different thicknesses (as shown in Figure 4b) was measured, and the results are also reproducible within experimental error. Comparison with the literature indicates that the measured values are close to the range of permeability characteristic for Pebax-1657, which varies in the available literature, namely *p*_CO2_ ~ 55–110 barrer and *α*(CO_2_/N_2_) ~ 40–70 [25,26,27,28,29,30].

CO_2_ and N_2_ permeability for different conditions/membranes and CO_2_/N_2_ selectivity were averaged for both polymers and results summarized in Table 1 and compared with the values from literature for similar materials. Permeability values obtained for thick membranes were used for double layer membrane gas permeability and permeance prediction using the resistance in the series model throughout the study (Equations (8) and (9)).

### 3.2. Resistance Model Predictions for Double Layer Pebax-1657/PDMS Membranes

**Figure 5:**
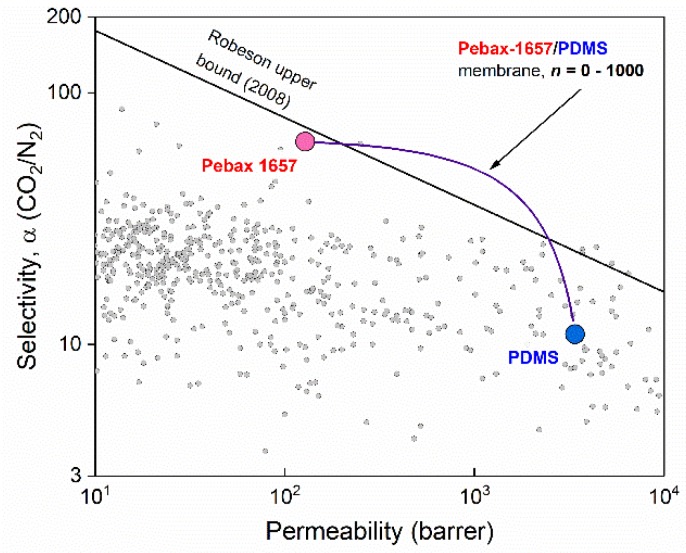
Resistance model predictions for the CO_2_ permeability of the composite membrane vs. CO_2_/N_2_ selectivity located in respect to the Robeson upper bound [4] and a number of the reported literature data (grey points) taken from the open database.

Using the experimentally measured permeability and selectivity values in Equations (8) and (9), we can predict the gas permeability behavior for composite membranes. As shown in the equations, the final permeability and selectivity are not dependent on the thickness of individual layers rather than the ratio of the thicknesses *n*. With the increase of the ratio, the permeability of the double layer membrane will be smoothly varied between its extreme values, namely, *p_t_* = *p_s_* when *n* = 0 and *p_t_* = *p_g_* when *n*→∞ as shown in Figure 5. It is interesting to note that the apparent permeability of the double layer membrane according to model prediction goes linearly from Pebax-1657 towards PDMS with the increase of *n*. This linear behaviour is the straightforward implication of the resistance model: resistances of two layers are simply added to each other. However, when the transition is depicted in usual log-log scale of Robeson plot it follows a complex path and breaks the Robeson upper bound [4], as the materials in the composite membrane share the properties at some values of *n* (namely from *n* ≈ 1 until *n* ≈ 65).

From the practical point of membrane application, it is more interesting to look not at the permeability values but the permeance of the composite membrane depending on the total thickness. Using the prediction of the gas permeability/selectivity performance of the Pebax-1657/PDMS membranes with different combinations in each layer (Figure 5), it seems possible to design membranes with selectivity close to the selectivity of Pebax-1657, but with much higher permeability. This could be used as a tool in the development of membranes desirable for their industrial post-combustion CO_2_ capture [3]. Resistance in the series model predicts a wide and realistic range of possible thickness combinations in the Pebax-1657/PDMS system (*n* values). The problem of the model in its simplified application, as performed here, is ignorance of the possible defects in the selective layer as well as porous support resistance, which indeed may become a significant factor as the gutter and selective layer become considerably thinner [5,31]. The purpose of further experiments is to check whether the resistance model predictions could serve as indicative for the planned membrane performance.

### 3.3. Cross Section Morphology of the Thin-Film Composite Membranes

**Figure 6:**
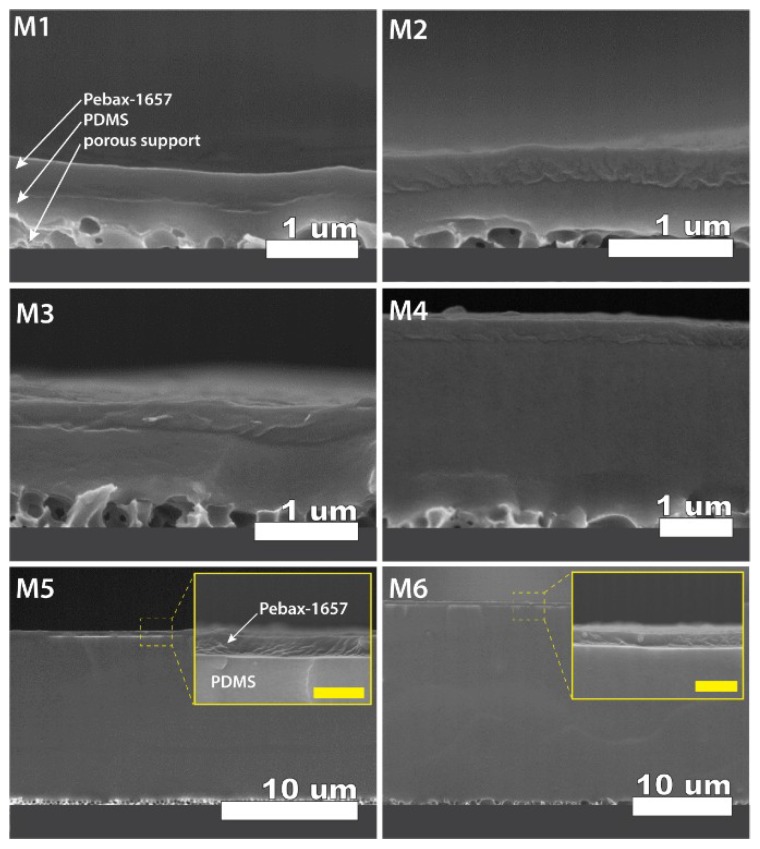
Cross-section scanning electron microscopy (SEM) images (acquired on Hitachi S-5200 FESEM, with accelerating voltage 5 kV) reflecting the morphology of different fabricated TFC membranes (codes indicated in each image). All membranes containing Pebax-1657 as selective (upper) layer, PDMS as a gutter layer (middle), and porous support (bottom) as indicated for membrane **M1**. Insets of the cross-section show magnified views of the top Pebax-1657 layer (yellow scale bars = 1 µm). Numerical values of the thicknesses and measurement errors can be found in Table 2.

To check the applicability of the resistance model predictions, we have fabricated the TFC membranes with different thicknesses of the PDMS layer. Cross-section scanning electron imaging was used to observe the morphology of the fabricated membranes and also to estimate the thickness of both the selective layer and gutter layer. As seen from Figure 6 we succeeded in fabricating the membranes with clearly defined morphology, and all layers were distinctively visible and well adhered to each other and the porous support. The unique combination of the materials and method used to fabricate the membrane enabled us to see the interface between the gutter layer and porous support. This was achieved exclusively thanks to the formation and crosslinking of the PDMS membrane on the solid support at first, and subsequent transfer onto the porous support. Such an approach enabled us to detect the clear interface between the PDMS layer and porous support in contrast to conventional depositions directly on the porous support [12,13].

Similarly, due to the different physical nature of the polymers, a clear interface is observed between the selective Pebax-1657 layer and PDMS. Therefore, acquired scanning electron microscopy (SEM) images can serve for both layers’ accurate thickness estimation. To obtain the distribution of thicknesses and standard deviation, the image analysis software (ImageJ 1.52d) was used to measure thickness in at least ten different places of the membrane. Table 2 summarizes the results of thicknesses for both layers. Subsequently, the thickness results were used to calculate model predictions for CO_2_ and N_2_ permeabilities, permeances, and CO_2_/N_2_ selectivity in fabricated membranes according to Equations (8) and (9).

### 3.4. Comparison of the Experimental Results with Resistance in Series Model Predictions

The results of expected CO_2_ permeability, permeance, and selectivity for the fabricated membranes are also given in Table 2. Considering the ratio of thicknesses (*n*) between the two layers, the values of permeability followed the expected trend, namely as the thickness of the PDMS layer became significantly larger compared to Pebax-1657 layer (*n* increases), permeability increased and approached the values closer to that of PDMS. At the same time, predicted selectivity behaved oppositely and decreased with the increase of the *n* value. Despite the significant differences of the total membrane thicknesses, permeances were not greatly different, as also shown in Table 2. It is expected there were implications from the two materials resistance to gas permeation. Namely, as Pebaxs relative resistance is ca. 30 times larger than PDMS resistance, the membrane M3 is expected to have the highest permeance due to the lowest thickness of Pebax-1657 layer. At the same time, it is expected to give a considerably higher selectivity value as the *n* value is small.

**Figure 7:**
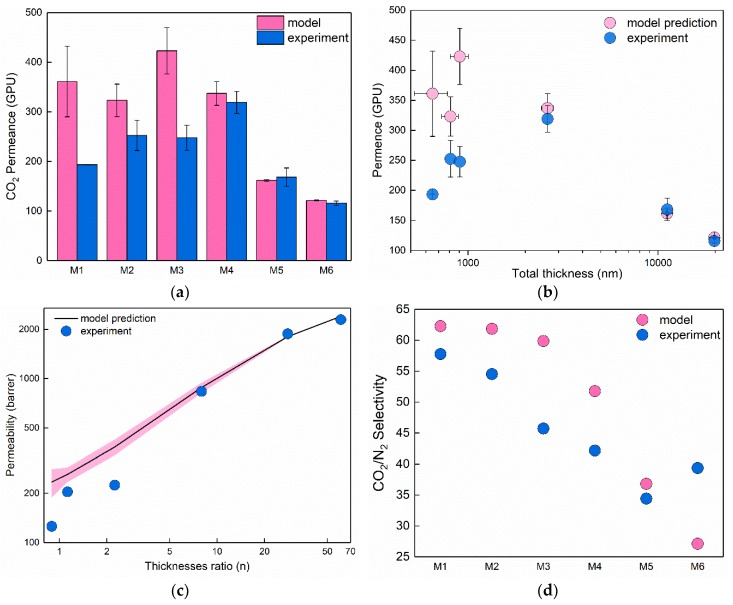
Comparison of the experimentally measured membrane performance with predictions calculated by resistance model: (**a**) CO_2_ permeance of fabricated membranes M1–M6; (**b**) CO_2_ permeance as a function of total membrane thickness; (**c**) CO_2_ permeability dependence on thickness ratio *n* (pink shading shows 95% confidence interval of prediction considering the error of thickness measurement); (**d**) CO_2_/N_2_ selectivity of fabricated membranes M1–M6.

All membranes were subjected to the actual pure N_2_ and CO_2_ gas permeation measurements. Figure 7 contains a comparison of the experimentally acquired permeances, permeabilities, and selectivity with the resistance model predictions. As seen from Figure 7a,b, three thicker membranes (M4–M6) demonstrated a perfect match with the resistance model prediction for CO_2_ permeances with the experimental data. In contrast, thinner membranes (M1–M3) demonstrated substantially lower permeances compared to what was predicted by the model (86%, 28%, and 70% lower respectively). The same effect, but to smaller extent was observed for N_2_ permeance, namely 73%, 13% and 30% decrease of permeances in thinner membranes. This behavior is also reflected in the total permeability of the membranes (Figure 7c), namely experimental data do not overlap with modelled data even considering the uncertainty induced with thickness measurements (pink shading around model prediction). The CO_2_/N_2_ selectivity calculated as a ratio of pure gas permeabilities compared to values predicted by the resistance model is shown in Figure 7d. It is seen that selectivity for most of the membranes is lower than expected by the model, which could be explained by the certain amount of pinhole defects in the Pebax layer. However, the decrease of selectivity in TFC membranes is relatively small compared to the decrements of permeance in thin membranes. Summarizing the results, we can conclude that resistance models provide quite accurate predictions for the membrane separation parameters, however, only in cases where total membrane thickness is larger than 2 µm (in this particular case at least). Membranes with total thickness below 2 µm demonstrated significantly lower permeances of both gases while the selectivity of all TFC membranes was not significantly altered (deviation <20%).

## 4. Discussion

Fabrication of the TFC membranes for industrial gas separation is a mainstream approach pursued by many research groups and companies. However, to the best of our knowledge, there are not many works systematically investigating the performance of TFC membranes in relation to the thicknesses of the layers. The problem with such an investigation often lies in the difficulties to fabricate layers with precisely defined thicknesses, especially when the polymer solutions are coated directly on the porous supports. For example, Qiao et al. used to coat PDMS directly on the polyacrylonitrile porous support to fabricate the layers with thicknesses below 300 nm [12,13]. Although they reported thin gutter layer of PDMS, both selectivity and permeability are significantly lower compared to the bulk, meaning that some forms of defects, pin holes, or surface pore clogging may exist. Most often the decreased values are explained by the fact that during a direct coating on the porous support, polymer solution penetrates to some extent into the pores of the support. For such layers, permeability–thickness–permeance relations are not fulfilled, e.g., 445 nm PDMS layer having a permeance of 2000 GPU and selectivity ~8 [12], which is much lower than expected if calculated from bulk PDMS permeability ~7000 GPU and selectivity ~11.6.

The principle difference of our approach is in how the membrane is fabricated and assembled. Namely, the PDMS layer is formed and cured, being attached to the flat solid support and not coated directly on the porous support. This guarantees that the film has uniform thickness and probability of defect formation is significantly suppressed. The selective layer is deposited on the smooth and defect-free PDMS, enabling similarly well-defined morphology and uniform thickness. The expectations from such a membrane fabrication approach are that our system should be more easily predicted by the resistance model as both layers have well-defined thickness. However, as is shown in the results, we still observed a very significant deviation of experimental data from the model predictions in the case of thinner membranes. Therefore, there may be some other factors causing this mismatch. One phenomenon that may add additional resistance into the fabricated membranes is the interfacial layer between PDMS and Pebax-1657, formed due to the ozone treatment that was used to activate the PDMS surface and promote the adhesion of the selective layer. Many researchers have reported the increased gas barrier of PDMS after surface activation by oxygen plasma [8,23] or ozone treatment [32]. In our work, we have intentionally chosen the ozone treatment as a milder activation process compared to O_2_-plasma treatment. However, if the effect is significant enough it would be seen on all membrane performances, which contradicts experimental results, i.e., permeance and permeability decrease being observed only for thinner membranes. Therefore, this influence cannot explain the gas separation in all fabricated samples.

Another plausible reason for the worsened mass transport through the fabricated membranes is the interaction with the porous support. The influence of porous support was recently modeled by several research groups using computational fluid dynamics (CFD) [31,33]. In particular, is was demonstrated by Ramon et al. [31] that with considerable thinning of the layer attached to the porous support, gas transport happens exclusively in the place of the open pore. Wijmans et al. have extended the CFD model onto the TFC membranes consisting of a gutter layer additionally to a selective layer [33]. Their results showed that as the thickness of the gutter layer decreases, the permeance starts to demonstrate a declining behavior, however, according to the calculations, this happens only in the range of small, “impractical” thickness ranges (below 10 nm). Although the effects of such ultimately thin layers are interesting, our membranes are far from the range where porous support is expected to have a dramatic impact on transport. If the influence is indeed present, membranes used in this work could demonstrate slightly lower permeances than expected. In fact, what has been observed is much stronger decline that can be seen by comparing the performance of M4 and M3 membranes in particular, where the latter has both thinner Pebax and PDMS layers (279 vs. 292 nm and 626 vs. 2327 nm respectively) but at the same time also demonstrates less CO_2_ permeance (248 vs. 319 GPU). Based on these speculations, we believe that it is unlikely that porous support has a determinative impact on the result.

Finally, one more reason for the lower permeances in thinner membranes could be related to the thickness itself. Plenty of research papers have reported the phenomena of low permeability in thin films due to accelerated aging, mainly in glassy polymer membranes [5,34,35]. Both polymers used in this work are, however, rubbery polymers (PDMS *T_g_*~−120 °C, Pebax MH1657 *T_g_*~−50°C) and are not susceptible to physical aging. The decreased permeability due to thickness itself was reported for PDMS [36], and similar behavior was reported for certain grades of poly(butylene terephthalate)-b-poly(ethylene oxide), which is known as Polyactive^®^ [7]. However, again the decrease that is reported is unlikely to be the single determining factor.

As a summary, we prepared the series of Pebax-1657/PDMS membranes with different thicknesses on a porous support and compared how the CO_2_/N_2_ separation performance corresponds with the predictions of resistance in series models. Surprisingly, lower permeances were observed in the thinner membranes compared with predictions made by the resistance model. We believe that deviation from the expected results can be caused by a cumulative effect from number of factors, such as ozone treatment influence, porous support influence, or thickness influence itself. Based on the current available data, it is difficult to conclude which factor is a primary contributor to the observed result. More detailed study is underway and will be reported elsewhere.

## Figures and Tables

**Table 1:** CO_2_ and N_2_ permeabilities and CO_2_/N_2_ selectivity of the bulk materials measured in this work compared to reported values in literature.

Material	CO_2_ Permeability, Barrer	N_2_ Permeability, Barrer	Ideal Selectivity	Reference
PDMS	3395 ± 55	307 ± 3	11	This work
	3100 ^a^	290	10.7	[23]
	3632 ^a^	330 ^c^	11	[24]
	3800 ± 70 ^b^	400 ± 10 ^b^	9.5	[21]
Pebax-1657	128 ± 17	2.0 ± 0.4	64	This work
	55.8	1.4	40.2	[25]
	80	1.1	70	[29]
	133	2.6 ^c^	52	[30]

^a^ Measured at 30 °C, ^b^ measured at 35 °C, ^c^ Calculated from permeability of CO_2_ and CO_2_/N_2_ selectivity.

**Table 2:** Parameters of the fabricated membranes and expected values of total CO_2_ permeability and CO_2_/N_2_ selectivity.

Membrane #	Thickness of Pebax-1657 Layer, nm (*l_s_ ±* Δ*l_s_*) ^1^	Thickness of PDMS Layer, nm (*l_g_ ±* Δ*l_g_*) ^1^	Ratio of Thicknesses, *n = l_g_/l_s_*	Model-Predicted CO_2_ Permeability, Barrer	Model-Predicted CO_2_ Permeance, GPU ^2^	Model-Predicted Selectivity
M1	343 ± 58	307 ± 70	0.90	235	361 ± 71	62.3
M2	380 ± 29	428 ± 53	1.13	261	323 ± 33	61.8
M3	279 ± 55	626 ± 45	2.24	383	432 ± 47	59.9
M4	292 ± 23	2327 ± 164	7.97	883	337 ±24	51.8
M5	385 ± 15	10,775 ± 90	27.99	1805	162 ± 2	36.8
M6	320 ± 15	19,469 ± 165	60.84	2403	121 ± 1	27.1

^1^ Errors Δ*l_s_* and Δ*l_g_* represent standard deviation of the ten independent measurements of thickness using SEM image analysis. Error for total thickness was calculated by addition of Δ*l_s_* and Δ*l_g_*. ^2^ Errors of model predicted permeances were calculated assuming relative error of total thickness equal to relative error of calculated permeance.
